# Mature dendritic cells enriched in immunoregulatory molecules (mregDCs): A novel population in the tumour microenvironment and immunotherapy target

**DOI:** 10.1002/ctm2.1199

**Published:** 2023-02-17

**Authors:** Jiaxin Li, Jun Zhou, Huanhuan Huang, Jiahuan Jiang, Ting Zhang, Chao Ni

**Affiliations:** ^1^ Department of Breast Surgery Second Affiliated Hospital Zhejiang University Hangzhou Zhejiang China; ^2^ Key Laboratory of Tumor Microenvironment and Immune Therapy of Zhejiang Province Second Affiliated Hospital, Zhejiang University Hangzhou Zhejiang China; ^3^ Cancer Center Zhejiang University Hangzhou Zhejiang China; ^4^ Department of Breast Surgery Affiliated Hangzhou First People's Hospital, Zhejiang University Hangzhou Zhejiang China; ^5^ Department of Radiotherapy Second Affiliated Hospital Zhejiang University School of Medicine Hangzhou Zhejiang China

**Keywords:** dendritic cells, tumour antigen‐presentation, tumour immune microenvironment, tumour immunology, vaccination

## Abstract

**Background:**

Dendritic cells (DCs) mediate divergent immune effects by activating T cells or negatively regulating the immune response to promote immune tolerance. They perform specific functions determined by their tissue distribution and maturation state. Traditionally, immature and semimature DCs were described to have immunosuppressive effects, leading to immune tolerance. Nonetheless, recent research has demonstrated that mature DCs can also suppress the immune response under certain circumstances.

**Main body:**

Mature DCs enriched in immunoregulatory molecules (mregDCs) have emerged as a regulatory module across species and tumour types. Indeed, the distinct roles of mregDCs in tumour immunotherapy have sparked the interest of researchers in the field of single‐cell omics. In particular, these regulatory cells were found to be associated with a positive response to immunotherapy and a favourable prognosis.

**Conclusion:**

Here, we provide a general overview of the latest and most notable advances and recent findings regarding the basic features and complex roles of mregDCs in nonmalignant diseases and the tumour microenvironment. We also emphasise the important clinical implications of mregDCs in tumours.

## Graphical abstract

- mregDCs are conserved across tumor types, diseases and species.
- The diversity roles of mregDCs in the tumor microenvironment are discussed.
- Summarizes the research exploration of mregDCs based on single cell analysis.
- Prognostic value and clinical implications of mregDCs in immunotherapy are emphasized.

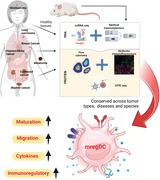

## 1 INTRODUCTION

Dendritic cells (DCs) are widely acknowledged as the most important and efficient antigen‐presenting cells (APCs) in the immune system. Within the tissue microenvironment, DCs orchestrate local antitumour immunity in various cancers. Even though they were discovered 50 years ago, their specific markers, subclassifications and relationships with other immune cells remain controversial.^1^ Detected in various tumour entities, DCs can be roughly divided into two main subsets: antigen‐presenting conventional DCs (cDCs) and interferon (IFN)‐producing plasmacytoid DCs (pDCs). cDCs are composed of two major branches: tumour‐associated antigens can be presented by cDC1s to cytotoxic CD8^+^ T lymphocytes, whereas cDC2 cluster cells are more focused on stimulating CD4^+^ T‐cell responses through major histocompatibility comlex (MHC) II.^2^ Apart from that, monocyte‐derived DCs (moDCs) are typically connected to inflammatory settings in both humans and mice, corresponding to effector T‐cell activity.^3^ moDCs profoundly influence tumour progression and are essential for the efficacy of anti‐programmed cell death‐1 (PD‐1) immunotherapy.^3^ Therefore, strategies for the precise manipulation of DCs could be of incredible value for increasing the efficacy of antitumour treatments.

Since single‐cell RNA sequencing (scRNA‐seq) technology has become widely available over the past few years, the field of DC phenotyping has gained considerable momentum. In this context, scRNA‐seq aids in unraveling the potential heterogeneity of DCs against the background of cancer and reveals new functions. An increasing number of studies have revealed other novel DC clusters based on the transcriptional program through fate mapping studies or transgenic animal models. AXL^+^SIGLEC6^+^DCs (AS‐DCs) are defined by the expression of surface markers AXL and SIGLEC6, and are related to both pDCs and cDC2s cells.^4^ Moreover, DC3s (CD1c^+^CD14^+^CD163^+^) are distinct from the classical cDC lineage, and these cells expand in the context of inflammatory diseases and exhibit an excellent potential to activate naive T cells into tissue‐resident memory T cells (Trm) that secrete IFN and tumour necrosis factor (TNF).^5^ To date, the lineage‐specific anti‐ and protumour activities of DCs in cancer biology have been the subject of numerous investigations. However, targeting various DC populations with conserved programs will likely help advance DC‐targeting cancer therapeutics. Thus, the programs relatively conserved across DC lineages during tumour development were investigated.

Recently, a conserved program has been widely depicted in multiple scRNA‐seq‐relevant studies on human and mouse cancer. Mature DCs enriched in immunoregulatory molecules (mregDCs) represent DCs with a typically conserved regulatory program and are considered a key element in the tumour microenvironment (TME).^6^ mregDCs have been detected in different studies under different titles, including Zilionis et al.’s^7^ ‘DC3’ cluster and Zhang et al.’s^8^ CCR7^+^LAMP3^+^ DCs. As a result, a proposal to universally refer to these cells as mregDCs was made to guarantee clarity when sharing related findings.^9^ In this timely review, we concentrate on summarising significant discoveries and the current understanding of the contribution of mregDCs to tumour biology and addressing their clinical implications to guide further research.

## 2 BASIC CHARACTERISTICS OF mregDCs

**FIGURE 1:**
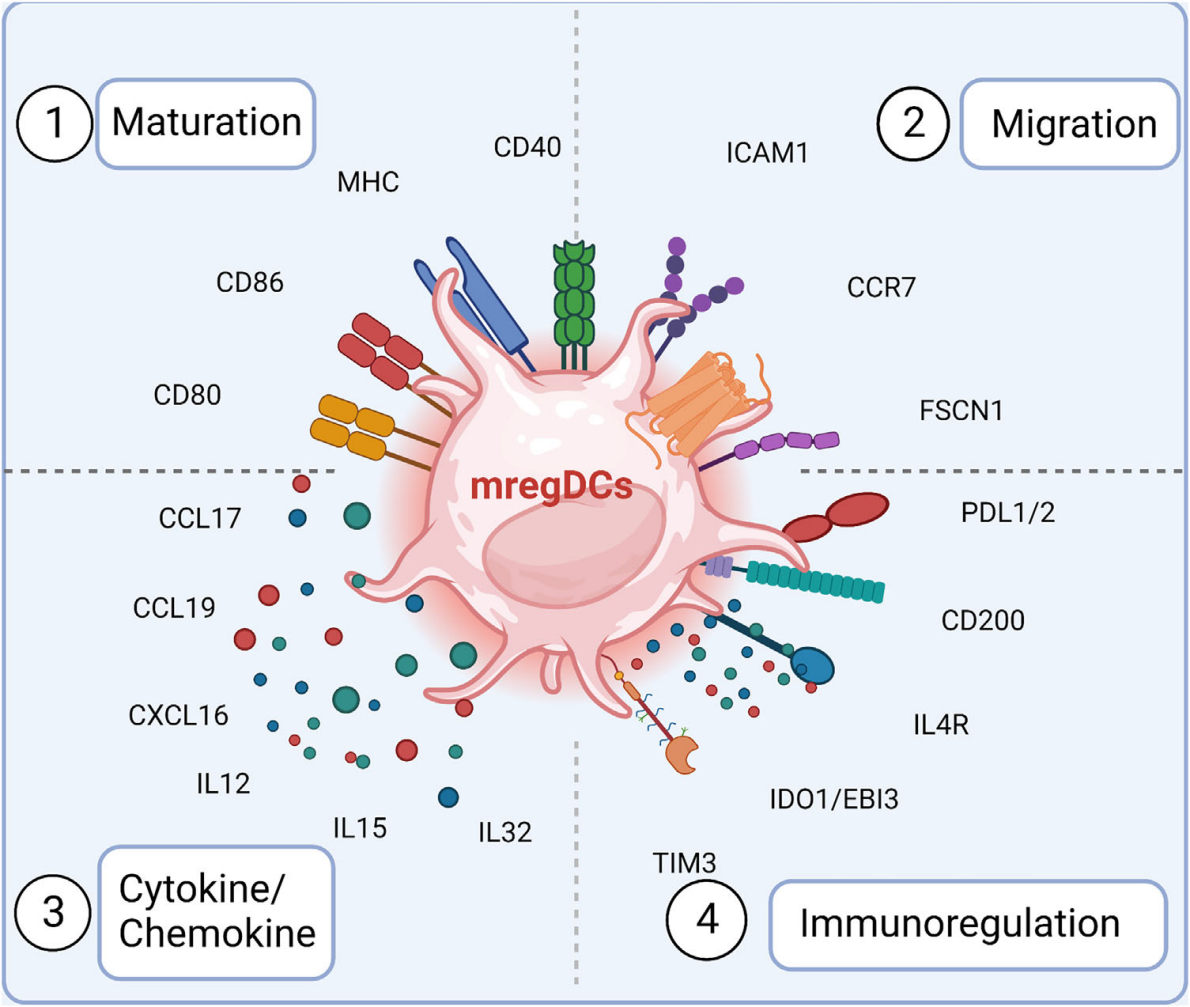
Basic characteristics of mature dendritic cells enriched in immunoregulatory molecules (mregDCs) in the tumour immune microenvironment. mregDCs are found in a wide range of tumours and tissues and are highly conserved among species. There are four distinct features of this population—(1) maturation: mregDCs express numerous maturation‐related signature genes, such as lysosomal‐associated membrane protein 3 (LAMP3), CD40, CD86, CD80 and CD83. (2) Migration: they highly express CC chemokine receptor 7 (CCR7), fascin actin‐bundling protein 1 (FSCN1) and intercellular adhenson molecule 1 (ICAM1). (3) Chemokine and cytokine release capability: these cells highly express chemokine ligands such as CC chemokine ligand 17 (CCL17), CCL19 and CCL22, which contribute to recruiting other immune cells expressing CCR4, CCR7 and CXCR3. (4) Significant immunomodulatory function: mregDCs show high expression of immune checkpoint‐related molecules and immunosuppressive genes including programmed death ligand 1 (PD‐L1), PD‐L2, CD200, T‐cell immunoglobulin and mucin‐containing molecule 3 (TIM‐3) and IDO1, which limit T‐cell activation. These features serve as the foundation for subsequent research on mregDCs, offering novel concepts and justifications for DC‐based cell therapy as well as new therapeutic options for tumours based on altering the immune microenvironment. Created with BioRender.com.

First identified in non‐small cell lung cancer (NSCLC) research, mregDCs were considered to be DCs presenting a regulatory program that restrains antitumour immunity and is conserved in humans and mice.^6^ In contrast to cDCs and pDCs, which have unique lineages, mregDCs represent a general molecular state of DCs. Since mregDCs were identified, multiple independent studies have revealed the presence of mregDCs in a broad range of cancer types, such as hepatocellular carcinoma (HCC),^10^ lung cancer, colon cancer,^11^ pancreatic cancer, melanoma,^12^ head and neck squamous cell carcinoma (HNSCC),^13^ bladder cancer, and breast cancer^14^ (Table 1). Although mregDCs are labelled differently among researchers, many studies have discovered that lysosomal‐associated membrane protein 3 (LAMP3) is a fundamental recognition marker for mregDCs and is coexpressed with the CC chemokine receptor 7 (CCR7) and programmed death ligand 1 (PD‐L1). These cells are characterised by their state of activation and maturation, their migration and immunoregulation abilities, and their lack of expression of key cDC1/2 and pDC gene markers, including XCR1 and CLEC9A for cDC1s, CD1C and FCER1A for cDC2s and CLEC4C for pDCs.^7^ This novel and unique group of DCs has several characteristics, as described below (Figure 1).

**TABLE 1:** Studies based on single‐cell sequencing that identified mature dendritic cell enriched in immunoregulatory molecules (mregDC) populations in various tumours.

Cancer type	Methodology	Samples	Species	Key markers	Key findings	Name	Ref.
Triple‐negative breast cancer	10× genomics	Tumours before and after anti‐PD‐1 therapy	Human	CD274, PDCD1LG2, CCR7, CCL19	PD‐L1 was only expressed on mregDCs, which are more frequent in responsive patients during treatment	mregDCs	^38^
Breast cancer	10× genomics	Paired biopsies of metastatic or primary tumour tissue and peripheral blood taken at baseline and during treatment	Human	LAMP3, CCR7, CCL19, IDO1, PD‐L1	mDCs may activate CD4–CXCL13 and CD8–CXCL13, and show relevance to anti‐PD‐L1 therapy	mDCs	^70^
Lung adenocarcinoma	10× genomics	Tumour samples and normal samples at adjacent and distant sites	Human	CCL12, CCR7, FSCN1, TNFSF9, LAMP3	The loss of inflammatory phenotypes by DCs occurred in early‐stage LUAD	DCs–LAMP3^hi^	^94^
Non‐small cell lung carcinoma	inDrop	Tumours	Human	FSCN1, LAMP3, CCL19, CCR7, CCL22, MARCKSL1	These cells are absent in the blood, lacking features of cDC1 and cDC2 clusters	hDC3 cluster	^7^
			Mouse	*Fscn1, Ccr7, Ccl12, Tnfrsf9, Sema7a, Stat4, Il12b*	These cells are conserved between human and mice	mDC3 cluster	
	10× genomics	Lung adenocarcinoma	Mouse	*Cd274, Pdcd1lg2, Cd200, Cd40, Ccr7, Il12b*	The mregDC program was first defined for the first time in the single‐cell transcriptome	mregDCs	^6^
	PIC‐seq	Treatment‐naive early NSCLC lesions	Human	FSCN1, CCL22, CCL19	mregDCs physically interact with CD4^+^PD‐1^+^CXCL13^+^ T cells and actively engage these cells upon PD‐1 blockade	mregDCs	^61^
Hepatocellular cancer	10× genomics and SMART‐seq2	Tumours, adjacent tissue, healthy tissue, ascites, blood	Human	LAMP3, CD80, CD83,CCL19, CCL21, CCR7	LAMP3^+^ DCs can migrate to lymph nodes.	LAMP3^+^DCs	^8^
Multiple cancers	10× genomics	Blood, Tumour and nontumour Sites	Human	FSCN1, LAMP3, CCR7	These cells have a variety of developmental origins and roles	LAMP3^+^DCs	^34^
	10× genomics	Tumour and adjacent nonmalignant tissue	Human	CCR7, CCL17, CCL19, LAMP3	Migratory cDCs originate from cDC2 cluster cells in tumours	Migratory cDCs	^20^
Multiple cancers	–	Data integrated from previous studies	Human	LAMP3, CCR7, FSCN1, BIRC3, CD83	The profiles of these cells are highly conserved across species, patients, and tumour types	‘DC3’ cluster	^95^
Colorectal cancer	10× genomics and Smart‐seq2	Metastatic CRC, nonmetastatic HCC, liver metastasised colorectal cancer	Human	LAMP3, CCR7, FSCN1, CCL19	Associated with primary colorectal tumour and mesenteric lymph node	LAMP3^+^DCs	^96^
Glioblastoma	10× genomics	Tumours, recurrent tumours without prior immunotherapy, recurrent tumours with neo‐anti‐PD‐1 therapy, blood	Human	CCR7, LAMP3, CD80	Neo‐anti‐PD‐1 increased migratory DC subsets and induced DC activation	mDCs	^29^
	10× genomics CITE‐seq	Tumours	Human	CCR7, LAMP3, SAMSN1, CD273, CCL19, FSCN1	The profiles of these cells are conserved in humans and mice	migDCs	^97^
			Mouse	*Ccr7*, *Lamp3*, *Samsn1*, *Cd200*, *Fscn1*, *Socs2*, *Ccl5*			
ESCC	10× genomics	ESCC tumour tissues and adjacent normal tissues	Human	IDO1, LAMP3, FSCN1	These cells are a significant contributor to the immunosuppressive ESCC microenvironment	tDCs	^17^
Ovarian cancer	10× genomics	Tumour ascites from tumour patients and tonsils from healthy patients	Human	CCR7, LAMP3, CCL19, MARCKSL1, CD83, IDO1	These cells affect the infiltration of cytotoxic CD8^+^ T cells	End‐stage moDCs/activated cDC2	^40^
Melanoma	10× genomics	Tumour metastases	Human	LAMP3, IDO1, CCL19	These cells present with an activated phenotype and prolongs patient survival	‘DC3’ cluster	^73^
Nasopharyngeal carcinoma	10× genomics	Nasopharyngeal carcinoma, healthy donors	Human	LAMP3, CCR7, FSCN1, CCL19	The interactions between mregDCs and T cells are extremely varied	‘DC3’ cluster	^98^
Bladder cancer	10× genomics	bladder tumour and adjacent normal tissues	Human	LAMP3, CCR7, CCL17, CCL19, CCL22	These cells recruit Tregs into TME via cytokines	LAMP3^+^ DCs	^26^
Health and disease	–	Multiple sites	Human	LAMP3, CCR7, CCL22	These cells are conserved in healthy and diseased samples	mregDCs	^99^

Abbreviations: CCL, CC chemokine ligand; CCR, CC chemokine receptor; IDO1, indoleamine 2,3‐dioxignase;mDC, mature DC; LUAD, lung adenocarcinoma; cDC, conventional DC; tDC, tolerance DC; migDC, migratory DC; ESCC, oesophageal squamous‐cell carcinoma; CRC, colorectal cancer; HCC, hepatocellular cancer; LAMP3, lysosomal‐associated membrane protein 3; moDC, monocyte‐derived DC; NSCLC, non‐small cell lung cancer; PD‐1, programmed cell death‐1; PD‐L1, programmed death ligand 1; PIC‐seq, RNA sequencing of physically interacting cells; TME, tumour microenvironment; Treg, regulatory T cell.

### 2.1 Activation and maturation state

Several studies based on scRNA‐seq have noted that mregDCs show the highest activity^15^ and have the largest number of ligands among all DC populations.^8^, ^16^, ^17^ Importantly, immune costimulatory genes, such as CD40, CD80 and CD86, are highly expressed by mregDCs in multiple types of tumours. The costimulatory molecule CD40 expressed on mature DCs can interact with CD40 ligands on naive T cells, augmenting T‐cell activation and promoting DC survival.^18^ Alternatively, mregDCs upregulate multiple genes that are enriched in pathways related to the active state, such as antigen processing and presentation, DC differentiation, cytokine‐mediated signal transduction, membrane trafficking and leucocyte activation, suggesting the multifunctionality of the mregDC population.^15^

Mature DCs upregulate T‐cell activation motifs to initiate adaptive antitumour immunity, including MHC molecules.^2^ The scRNA‐seq analysis revealed that mregDCs exhibited the highest degree of differentiation by trajectory mapping,^16^ indicating the mature status of mregDCs. Similar to mature DCs, mregDCs displayed the highest amounts of MHC class protein and LAMP3 in all DC clusters.^6^ Moreover, as an important marker of DC maturation, LAMP3 plays an essential role in the process of exogenous antigen presentation to T lymphocytes in DCs^19^ and is not expressed on naive primary cDC1s, cDC2s or pDCs.^8^ Another recent study on the pancancer microenvironment reported that LAMP3 was only upregulated in mregDCs.^20^ Thus, DCs expressing LAMP3 may represent ‘true’ mregDCs, and this marker may assist in distinguishing them from other DC subpopulation states.

### 2.2 Migration capability

As the sentinel cells of the immune system, DCs are widely distributed throughout organs and many nonlymphoid tissues. Another characteristic of mregDCs is their ability to migrate to lymph nodes or tertiary lymphoid structures (TLSs), which allows them to effectively initiate antitumour immunity. LAMP3‐expressing mregDCs are more enriched in tumour‐draining lymph nodes (dLNs) and peritumoural clusters of T cells than in the primary tumour itself in melanoma,^21^ and this phenomenon was further verified in lung adenocarcinoma^22^ and breast cancer.^23^ In addition, mregDCs exhibited the strongest ability to migrate to the lymph node among all DCs. In a single‐cell transcriptome study of HCC, investigators confirmed by RNA velocity that mregDCs in tumours and dLNs share the same lineage.^8^ Furthermore, CD8^+^ T lymphocytes in dLNs share an identical T‐cell receptor with CD8+ T cells in primary tumours, which confirms that these T cells are primed by mregDCs that migrate from the primary tumour to the dLNs.^8^ Through interactions with CC chemokine ligand 21 (CCL21), which is generated by lymphatic endothelial cells,^24^ CCR7 promotes the mregDCs to migrate from primary tumours to lymph nodes.^7^ As assessed by flow cytometry, mregDCs were shown to express higher CCR7 levels than other DCs.^8^ According to another study, DCs were reprogrammed metabolically towards glycolysis in response to CCR7 stimulation, which promoted the hypoxia‐inducible factor‐1 (HIF‐1) pathway and facilitated DC migration.^25^ Furthermore, using a transwell migration assay, CD40 L/PGE2 or DC maturation kits could induce DCs to be more prone to migrate towards CCL19.^8^

### 2.3 Chemokines and cytokines

Consistent with the mature phenotype, mregDCs exhibit an increased release of cytokines and chemokines. CCL19, CCL17 and CCL22 are highly expressed on mregDCs, and they can recruit other lymphocytes expressing CCR7, CCR4 and CCR3.^26^, ^27^ Additionally, mregDCs are the predominant source of CCL17 and CCL22 in all APCs of HNSCC tissues.^28^ In addition, interleukin (IL)‐10, IL‐4 and IL‐35 are also highly expressed in mregDCs.^15^, ^29^ According to single‐cell investigations in lung cancer, mregDCs can release IL‐15 and promote tissue‐resident T‐cell function.^30^ In HCC, the upregulation of the STING signalling in mregDCs results in CXCL9 and IL‐12 secretion.^31^ Di Pilato et al.^32^ similarly noted that mregDCs in the perivascular region are also capable of secreting CXCL16 and the cytokine IL‐15. These cytokines and chemokines endow mregDCs with robust cellular communication capabilities to remodel the TME by recruiting or modulating a wide range of immune cells. Therefore, mregDCs can remarkably influence the TME and regulate tumour progression.

### 2.4 Immunoregulatory molecules

Immunomodulatory function is one of the most distinctive features of mregDCs. Several studies have demonstrated that mregDCs have the greatest amounts of immune checkpoint transcripts among all DCs. In a scRNA‐seq study of ESCC, Zhang et al.^17^ found that indoleamine 2,3‐dioxigenase 1 (IDO1), PD‐L1 and PD‐L2 were abundantly expressed in mregDCs and induced regulatory T‐cell (Treg) production. Similarly, flow cytometry and multicolour immunofluorescence confirmed the significantly increased expression of PD‐L1 in mregDCs.^15^ Previous research has demonstrated that PD‐L1, which acts as an immune suppressor when combined with PD‐1, is mainly derived from tumour cells. However, a recent paper indicated that macrophages and DCs (rather than tumour cells) are the predominant sources of PD‐L1 bound to PD‐1^+^ T cells.^13^ When analysing immune cells from bladder cancer tissues in a single‐cell transcriptome study, Chen et al.^26^ noted that PD‐L1 was maximally expressed in mregDCs, at an even higher level than in Tregs. Importantly, immunohistochemistry also confirmed that mregDCs uniquely express PD‐L1 within DCs in breast cancer.^26^ The high level of PD‐L1 expression was modulated by CMTM6, which was also upregulated in mregDCs in tumours.^33^ CMTM6 is a key PD‐L1 protein regulator, and it increases PD‐L1 expression on mregDCs by reducing PD‐L1 ubiquitination and prolonging its half‐life.^34^ These findings indicate that PD‐L1 is another broad marker for mregDCs, which shape the immunosuppressive ecosystem in tumours, especially in T‐cell dysfunction.^35^ Beyond the prevalent PD‐L1/PD‐L2 genes, other immunosuppressive genes, such as CD200, EBI3, SOCS1, SOCS2, SOCS3^16^ and LGALS9,^36^ have also been identified to be overexpressed. These genes could extensively limit the effects of T cells in multiple ways, impeding antitumour immune effects. Alternatively, T‐cell immunoglobulin and mucin‐containing molecule 3 (TIM‐3) is emerging as a novel immune checkpoint molecule. Although widely overexpressed on most DCs and macrophages, TIM‐3 is most abundantly expressed on mregDCs.^37^

## 3 MATURE DYNAMICS OF mregDCs

**FIGURE 2:**
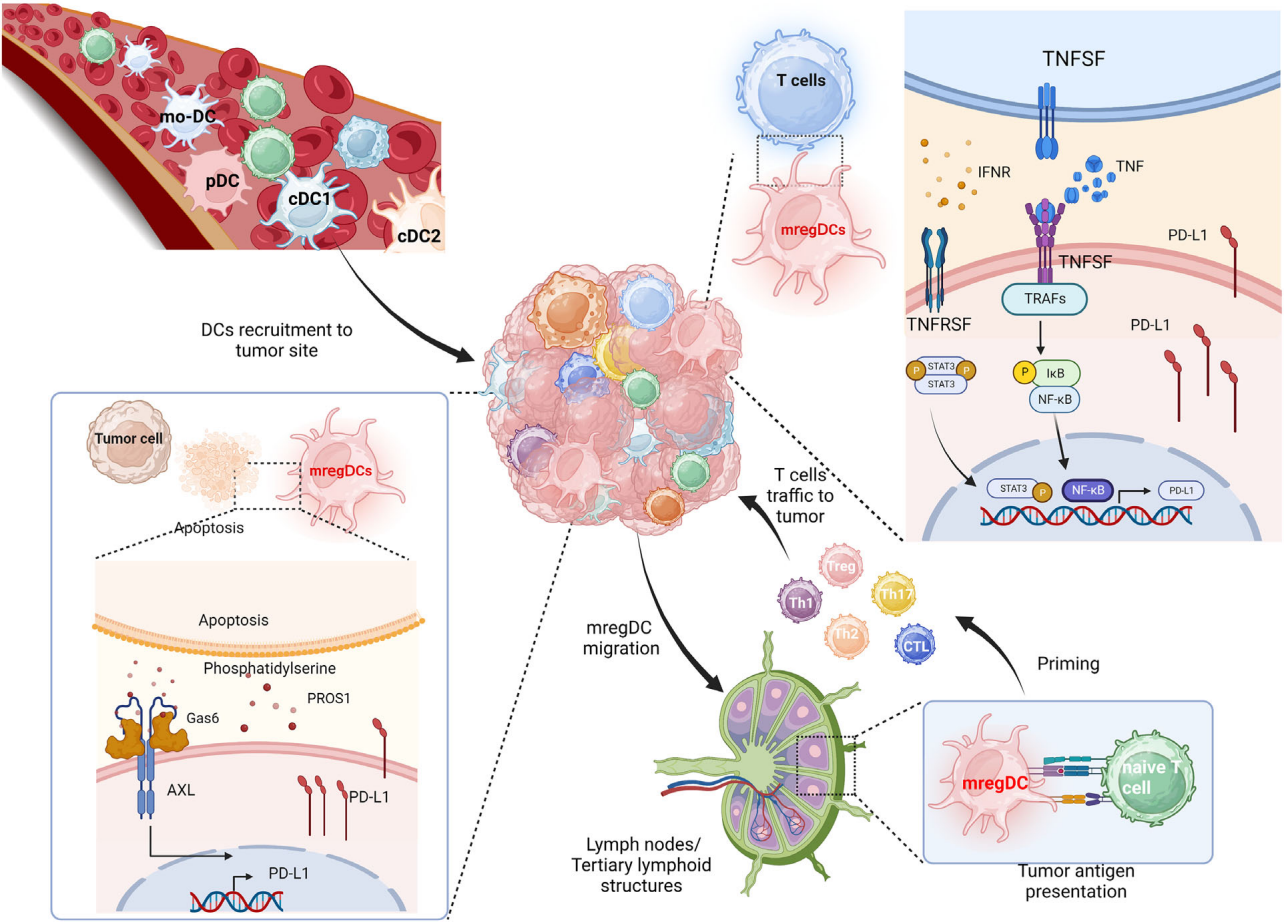
The dynamic maturation process of dendritic cells (DCs) with the mature DCs enriched in immunoregulatory molecules (mregDC) program. Different DC lineages—conventional DCs (cDC1 and cDC2), plasmacytoid DCs (pDCs) and monocyte‐derived DCs (moDCs)—can contribute to mregDCs. Following circulation, these terminally differentiated cells are recruited and arrive at the tumour site. After stimulation by tumour antigens, these naive DCs are activated, mature and initiate the mregDC program and migrate from the tumour to the peripheral lymph nodes or tertiary lymphoid structures (TLSs). mregDCs are regulated by a variety of cells in the tumour microenvironment. Apoptotic tumour cells release growth arrest‐specific protein 6 (Gas6) and protein S 1 (PROS1), which bind to the anexelekto (AXL) of mregDCs, initiating the mregDC program and leading to an upregulation of programmed death ligand 1 (PD‐L1) transcript levels in mregDCs. In addition, tumour necrosis factor (TNF) and interferon‐γ (IFNR) released by other immune cells activate the nuclear factor kappa‐B (NF‐κB) and the Janus kinase‐signal transducer and activator of transcription (JAK‐STAT) pathways, respectively, which promoting PD‐L1 transcription in mregDCs. Created with BioRender.com.

Under normal conditions, DCs exist in a steady state to perform their role as sentinel cells. However, upon uptake of tumour antigens, primary DCs are rapidly activated and undergo antigen presentation processing to transform to a mature state while being modulated by the tumour immune suppressive microenvironment, leading to the acquisition of the mregDC phenotype (Figure 2).^38^

It has been reported that many DC populations can be induced to express specific markers of mregDCs. Recently, the transcriptomes of peripheral cDC1s and cDC2s have been found to converge upon cancer‐induced maturation and acquire the mregDC program.^16^ The cDC1s and cDC2s that migrated to the lymph nodes also exhibited mregDC features, according to bulk transcriptomic analysis.^39^ Given that mregDCs lack distinguished markers of cDC1s and cDC2s at the transcriptome level, Pombo utilised cellular indexing of transcriptomes and epitopes by sequencing (CITE‐seq) technology to provide protein information on the distribution of mregDCs at single‐cell resolution.^38^ They revealed that cDC1s and cDC2s both made a significant contribution to mregDCs in humans and mice. However, mregDCs derived from cDC1s and cDC2s seem to have different features. A pancancer analysis noted that cDC1‐like mregDCs are more common in the majority of tumours, while cDC2‐like mregDCs are prone to be present in pancreatic and nasopharyngeal cancers.^34^ In an experimental model, mregDCs were also found in pDC subsets in hepatocellular cancer, suggesting that the mregDC program could exist in pDCs.^08^ Similarly, the transcriptional profiles of moDCs^40^ and AXL^+^SIGLEC6^+^DCs^41^ could also converge into the mregDC state. The results taken together imply that all human DC clusters might display the mregDC transcriptional program under specific circumstances, which is in line with earlier research indicating that all types of DCs undergo profound and convergent transcriptional changes during their maturation.^42^

To the best of our knowledge, an intricate web of cytokines and other variables may have an impact on the mregDC polarisation program. From the results of in vitro cellular experiments, it appears that DCs initiate the mregDC program when costimulatory and inhibitory signals cooccur. When exposed to CD40 L and prostaglandin E2 (PGE2), moDCs could present a quintessential mregDC state with an increased level of LAMP3 and migration to CCL19.^8^ IFNγ and lipopolysaccharide (LPS) were also reported to be critical regulators of mregDCs. Upon in vitro stimulation with LPS^+^ and IFNγ, CD83, LAMP3, PD‐L1 and IDO were all upregulated in DCs, and these markers are key to the mregDC program.^15^ In addition, tumour cells typically remodel the TME by metabolising elements and show the potential to regulate the mregDC program. The hypoxic environment leads to an increase in hypoxia‐inducible factor‐alpha (HIF‐α) levels in tumour cells, which diminishes the ability of mregDCs to present antigens in the quiescent cancer cell niche.^43^ This suggests the strategy of applying HIF inhibitors to restore the antigen‐presenting ability of DCs.^43^ Nikolos et al.^44^ also discovered that releasing PGE2 from tumour cells hindered the maturation of DCs and promoted the mregDC program.

Although previous reports demonstrated that DC subsets could be reprogrammed into mregDCs with various stimuli, the specific transcriptomic profiles of mregDCs support their diverse functional properties. CITE‐seq revealed that cDC1‐like mregDCs expressed high levels of IL‐12B, CCL17, IRF8 and CADM1 at both the RNA and protein levels. IL‐12B is specifically expressed in cDC1‐like mregDCs and is capable of inducing the differentiation of T helper 1 (Th1) cells,^34^ indicating that the lineage‐specific profiles of DC subpopulations are conserved in the mregDC state.^42^ Furthermore, according to a recent study, cDC1‐derived mregDCs play the dual roles in regulating both CD8^+^ T cells and Tregs,^17^ which is line with the intricate coexpression profile of activating and inhibitory molecules of cDC1‐like mregDCs.^34^ In addition, research found that patients with colorectal cancer who had more activated cDC1s, which may correspond to cDC1‐like mregDCs, had a favourable prognosis.^32^ In contrast, cDC2‐like mregDCs highly expressed the cDC2 marker gene CD1E together with SIRPA and FCER1G^6^ and showed CXCL9 deregulation and IDO1 upregulation,^10^ representing enhanced immunosuppressive functionality. The migration function indicates that mregDCs can migrate to other sites to perform antigen‐presenting functions after being stimulated by antigens, yielding a broad immunomodulatory effect.

## 4 mregDCs IN THE NONTUMOUR MICROENVIRONMENT

With research progress, many academics have also found the existence of mregDCs in a variety of normal tissues and disease‐related tissues (Table 2). In a single‐cell study, researchers surprisingly confirmed the existence of mregDCs in healthy corneas in adults.^45^ Furthermore, they found that mregDCs were also present in the skin, and mregDCs in both tissue types exhibited high expression of CCR7, CD274 and IDO2. However, mregDCs only make up a minor part of the skin's overall DC population. All corneal DCs were identified as the mregDCs.^45^ mregDCs in the cornea function as APCs and may take part in the control of immunological tolerance through collaborating with other immune cells.^45^ Tonsils are an important part of our immune system that impede bacteria from entering the body through the mouth and nose. mregDCs are also found in tonsils and express LAMP3 and CCR7.^40^ When compared to those in inflamed skin, mregDCs in the tonsils exhibit higher expression of RELB, IER2 and SPI1.^46^ mregDCs have also been identified in the thymus and play a significant role in generating Tregs. In addition to transcriptomic analysis, RNA in situ single‐molecule fluorescence in situ hybridisation showed the colocalisation of mregDCs and Tregs,^47^ indicating their spatial relationship.

**TABLE 2:** Mature dendritic cells enriched in immunoregulatory molecules (mregDCs) in the inflammatory microenvironment in single‐cell transcriptome research.

Disease	Methodology	Samples	Species	Key markers	Name	Ref.
Acute sterile skin inflammation	scRNA‐seq	Healthy skin and sterile skin wounds	Human	CD83, CCL22, CCR7, HLA‐DPA1 and LAMP3	BDCA‐2^+^ CD123 int DCs	^41^
Chronic inflammatory skin diseases	Smart‐seq2	Healthy skin and atopic dermatitis and psoriasis lesions	Human	CCL17, LAMP3, BIRC3, CD200, IL‐15, IL‐32	mregDCs	^46^
Atopic dermatitis	10× genomics	Lesional and nonlesional skin	Human	CCR7, CCL22, LAMP3, BIRC3	LAMP3^+^CCR7^+^ DCs	^48^
Polymicrobial sepsis	10× genomics	Bone marrow, peripheral blood and spleen	Mouse	*Cd86*, *Cd274*, *Marcks*, *Il4ra*, *Ccr7*	mregDCs	^49^
Atherosclerosis	Public datasets	Mouse aortic leucocyte	Mouse	*Fscn1*, *Ccr7*	mregDCs	^100^
–	10× genomics	Healthy corneal tissue	Human	LAMP3, BIRC3	mregDCs	^45^
Oropharyngeal diseases	10× genomics	Tumour tissues and nonmalignant inflamed tissues	Human	LAMP3, CCR7, CCL19, CSF2RA	mregDCs	^28^

Abbreviations: CCL, CC chemokine ligand; CCR, CC chemokine receptor; IL, interleukin; LAMP3, lysosomal‐associated membrane protein 3; scRNA‐seq, single‐cell RNA sequencing.

**FIGURE 3:**
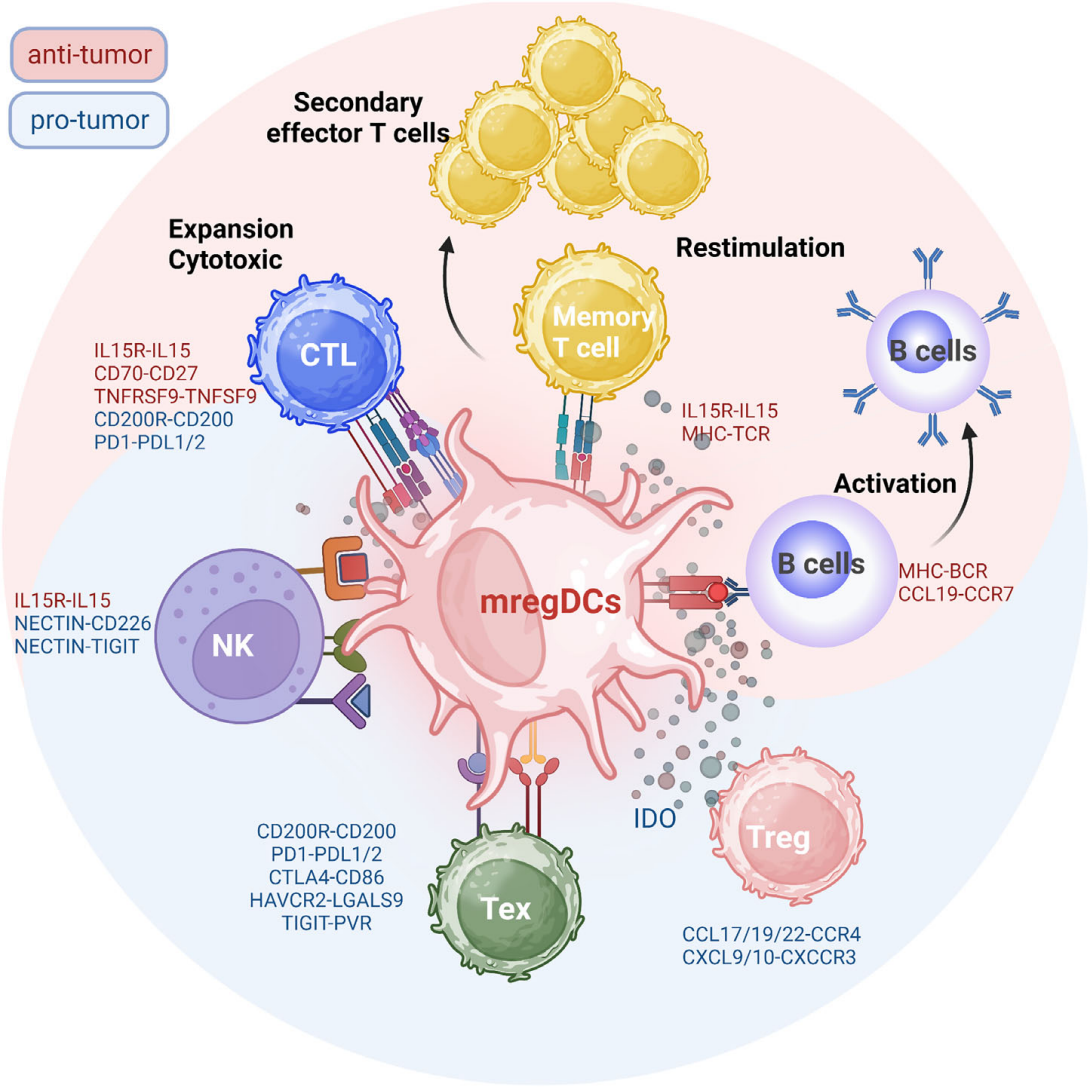
Interactions of mature dendritic cells enriched in immunoregulatory molecules (mregDCs) with other lymphocytes in tumour immune microenvironment. Multiple ligand genes are expressed by mregDCs, allowing them to interact with a wide variety of T cells. In this figure, the pink area represents the antitumour immunity induced by mregDCs, and the blue denotes their immunomodulatory function. The immunosuppressive function of mregDCs can result in T‐cell exhaustion by immune checkpoint molecules and recruit regulatory T cells (Tregs) through secreted chemokines forming an immunosuppressive microenvironment. Maturation of DCs presenting the mregDC program and antigen‐presenting functions in turn confer antitumour capacity, stimulating B cell and T lymphocyte activation through the presentation of tumour antigens and expression of immune costimulatory molecules. mregDCs play a crucial and irreplaceable role in the immune microenvironment. Created with BioRender.com.

Inflammation is acknowledged as one of the hallmarks of cancer and is not unique to tumours. Many investigators have found mregDCs in patients with inflammatory diseases, and their presence and functionality are closely related to the disease state. mregDCs were first recognised in human skin by Chen et al.^41^ in the context of acute aseptic skin inflammation and were defined as activated DCs. High expression of DC maturation‐related genes, including HLA‐DPA1 and LAMP3, was one of their distinguishing features. This group of cells infiltrated near the injured skin at the early stage of wound formation and underwent rapid renewal, contributing to the formation of the skin's immune network and thus assisting in wound healing.^41^ mregDCs also play a crucial function in chronic inflammatory disorders. mregDCs (LAMP3^+^CCR7^+^DCs) exhibit significantly higher infiltration in skin affected by atopic dermatitis (AD) than in healthy skin.^48^ This finding was confirmed by another work, which also identified mregDCs as a major source of IL‐15 in AD and psoriasis (PSO). These cells drive the production of Th17 cells and play a key role in the pathogenesis and recurrence of chronic inflammatory skin diseases. Therefore, modulation of mregDC function and cytokine secretion with targeted drugs might contribute to alleviate the AD and PSO.^48^ The mregDC program can also be driven by sepsis and is tightly associated with hyperinflammatory stages.^49^ To identify differences in mregDCs in the tumour versus inflammatory condition, Mair et al.^28^ compared the single‐cell transcriptome of mregDCs in inflamed tissue of the oral mucosa and in oral squamous carcinoma. They specified that most of the mregDC phenotypes in both states, including the high expression of PD‐L1, were essentially identical, which may explain the inflammatory side effects that sometimes occur with systemic anti‐PD‐L1 therapy (Figure 3).

## 5 THE COMPLEX CROSSTALK BETWEEN mregDCs AND THE TME

### 5.1 mregDCs suppress the immune response by regulating immune checkpoint molecules

Immune checkpoint molecules have been found to be widely expressed on mregDCs, and extensive scRNA‐seq studies have made it possible to comprehensively understand the ligand–receptor interactions between mregDCs and other cells. Paired ligand–receptor analyses suggest that mregDCs are more likely to regulate exhausted CD8^+^ T cells and Tregs via PD‐1/PD‐L1 in HCC.^8^ This pattern was also observed in the study of oesophageal cancer and was further validated by coculture experiments. Following the coculture of naive CD8^+^ T cells with tumour‐derived mregDCs, the proliferation and effectiveness of CD8^+^ T cells were significantly curtailed by PD‐1/PD‐L1 interactions.^17^ PD‐1/PD‐L1, CTLA4–CD86 and LGALS9–HAVCR2 interactions among mregDCs and various T cells were widely observed in colorectal tumours, promoting Tregs and dysfunctional T cells and hindering the effective immune response.^36^ In addition, a nonclassical immunosuppressive pathway engaged in suppressing antitumour responses, CD200‐CD200R signalling, was also predicted to be an important way for mregDCs to interact with CD8^+^ T cells.^16^ In mycosis fungoides tumours, the expression of PVR was revealed to be extremely high in mregDCs.^50^ The poliovirus receptor (PVR) on mregDCs has a high affinity for TIGIT expressed on effector natural killer (NK) cells or CD8^+^ T cells, and the PVR–TIGIT signalling axis is known to have a strong immunosuppressive effect. Furthermore, mregDCs can also directly modulate liver‐resident NK cells via the NECTIN–TIGIT interaction, delivering an inhibitory signal.^8^

Another unique approach to forming an immunosuppressive microenvironment in mregDCs is prolonged and sustained stimulation, which induces T‐cell exhaustion programs. From a conventional perspective, DCs and macrophages contribute to the rapid differentiation of T cells and yield effective antitumour functions by presenting tumour antigens. However, a recent study in melanoma showed that cDC2‐like mregDCs contribute to only CD4^+^ T‐cell initiating activation rather than subsequent differentiation, consequently causing a defect in antitumour effector T cells. These results indicate that mregDCs could lead to impaired immunity by inhibiting CD8^+^ T cells directly or by inducing T‐cell dysfunction.^51^ This phenomenon was also reported in tumour‐associated macrophages (TAMs). Recent work has demonstrated that augmented antigen presentation by TAMs cannot completely support the infiltration and function of CD8^+^ T cells but instead initiates T‐cell exhaustion programs through persistent antigen‐specific synaptic interactions.^52^ Moreover, the physical interactions of mregDCs with PD‐1^+^ cytotoxic T lymphocytes (CTLs) and PD‐L1^+^ macrophages were imaged by 3D high‐resolution microscopy. When tumours become locally invasive, they participate in the formation of a reinforced, spatially restricted immunosuppressive environment alongside the tumour–stroma border and facilitate tumour survival.^11^ Therefore, mregDCs contribute to immunosuppression through direct contact in two ways. On the one hand, mregDCs suppress the T‐cell response by expressing ligands for various immune checkpoints; on the other hand, they can provoke T‐cell exhaustion through direct chronic antigen presentation stimulation, impairing T‐cell antitumour immune function.

### 5.2 mregDCs support the immunosuppressive microenvironment by secreting cytokines/chemokines

mregDCs are predominantly enriched in tumour tissues compared to adjacent noncancerous tissues in various cancers as revealed through imaging mass cytometry and single‐cell resolution analysis.^8^, ^15^, ^34^ In the context of the TME, mregDCs extend their immunosuppressive function by secreting cytokines and chemokines. Notably, mregDCs express CCL17, CCL19 and CCL22,^26^, ^27^ which promote Treg migration into the TME by binding to CCR4 and promoting tumour invasion and drug resistance to immune checkpoint blockade (ICB) treatment.^53^ In the bladder cancer cohort, the mregDC signature correlated strongly with the Treg and Th2 signatures but not with the CTL signature. It is worth highlighting that both Treg and Th2 cells were identified as CCR4‐positive cells.^26^ In addition, in silico model predictions in the human thymus indicated that CXCR3‐expressing pDCs and mature Tregs are in turn likely to be recruited by mregDCs via CXCL9/10‐CXCR3. Furthermore, coexpansion of Tregs and cDC2‐like mregDCs results in tumour immune tolerance, while therapeutic depletion of Tregs effectively augments cDC2 maturation and reverses the phenotypic dysfunction, thereby facilitating the production of antitumour CD4^+^ T cells.^51^

In addition, IDO1 is highly expressed in mregDCs across an array of human tumours,^17^ which might enable the identification of tumour‐specific mregDCs. DC‐expressed IDO1 contributes to the differentiation of Tregs while simultaneously inhibits CD8^+^ T‐cell proliferation and effector functions as well as NK cell and plasma cell proliferation.^54^ Furthermore, PD‐L1 and IDO expression in mregDCs enhanced the ability of mregDCs to induce Treg differentiation when cocultured with CD4^+^CD45RA^+^ naive T cells.^15^ In addition, IL‐10, IL‐4 and IL‐35 are also highly overexpressed in mregDCs,^15^, ^29^ which effectively promotes Treg transition and restrains the antitumour function of CD8^+^ T and CD49^+^ NK cells. Furthermore, scRNA‐seq analysis of nasopharyngeal carcinoma indicated that the overexpression of suppressor of cytokine signalling (SOCS)‐related genes may also be an important pathway by which mregDCs exert their suppressive antitumour function.^16^ These findings suggest that relevant cytokine blockers may help restore antitumour T‐cell responses by targeting mregDCs.

### 5.3 Antitumour activity of mregDCs

mregDCs have higher activity than other DC subsets and act as the hub of the immune system. Increasing evidence has also demonstrated that mregDCs can exert antitumour immunity by activating primitive T cells for antigen presentation and interacting extensively with B cells and NK cells. Consistent with these speculations, mregDCs were shown to be capable of interacting with CD8^+^ T cells via TNFRSF9–TNFSF9, offering a strong costimulatory signal to facilitate efficient cytotoxic CD8^+^ T‐cell differentiation.^17^ In addition, mregDCs express high levels of the costimulatory signal CD70, which is involved in the differentiation of cytotoxic CD8^+^ T cells via CD27.^38^, ^40^ Moreover, according to a single‐cell study of lung cancer, Trm expansion and homeostatic survival may be facilitated by mregDCs through IL‐15 and IL‐15R binding,^30^ which are vital cytotoxic immune cells and are responsible for the effects of immunotherapy. Di Pilato et al.^32^ also noted that CXCL16 and IL‐15 support the activation of CXCR6‐expressing tumour‐invasive CTLs by mregDCs in the perivascular niche of the TME. Therefore, mregDCs provide crucial survival and proliferation signals to local T cells to maximise their antitumour activity.

In addition, mregDCs also enhance innate antitumour immunity through modulation and enhancement of NK‐cell activity. By assessing ligand–receptor pairs, it was anticipated that mregDCs would interact with NK cells via NECTIN2–CD226 and confer an activating signal.^8^ Moreover, IL‐15 from mregDCs also promotes the proliferation of NK cells and enhances their antitumour function. According to a mouse model of HCC, researchers discovered that the administration of CD47 blockade promoted the infiltration of both mregDCs and NK cells, and the activation of STING in mregDCs led to CXCL9 and IL‐12 secretion, further promoting the infiltration and activation of NK cells.^31^

In addition to conferring direct antitumour effects, mregDCs (LAMP3^+^ DCs) are an essential component of TLSs in human tumours.^33^, ^55^ In regions of the tumour stroma, especially at the boundaries around the tumour, mregDCs are often detected in clusters with T cells, characteristic of a sustained immune response.^56^, ^57^ Organised TLSs provide an immune‐supportive niche for quick surveillance, which is associated with increased patient survival in various tumours.^58^, ^59^ In lung cancer, mregDCs are localised in TLSs and close to T cells, consistent with their roles in T‐cell activation and clonal expansion.^60^ In addition, consecutive immunohistochemical staining of multiple markers on a single slide showed that LAMP3‐ and PD‐L1‐expressing mregDCs accumulated in TLSs close to T cells.^27^ Recent research has highlighted that microaggregates composed of CD8^+^ (CD103^+^) T cells, CD4^+^ T cells and mregDCs within tumour cell beds in oropharyngeal squamous cell carcinoma have remarkable similarities with TLSs. They constitute as a positive feedback loop to maintain the formation of DC–T‐cell microaggregates and identify patients with unprecedented survival rates after standard therapy.^60^ Additionally, Cohen et al.^61^ developed a novel technology to show the preferential interactions between PD‐1^+^CXCL13^+^ helper T cells (Th cells) and mregDCs in the TME by RNA sequencing of physically interacting cells. In addition, this finding was confirmed by confocal microscopy images of TLSs, which a marker of a vigorous antigen‐specific adaptive antitumour immune response that confers a prognostic benefit in anti‐PD‐L1 treatment for patients with NSCLC,^27^ demonstrating the high value of mregDCs in regulating the immunotherapy response.

**FIGURE 4:**
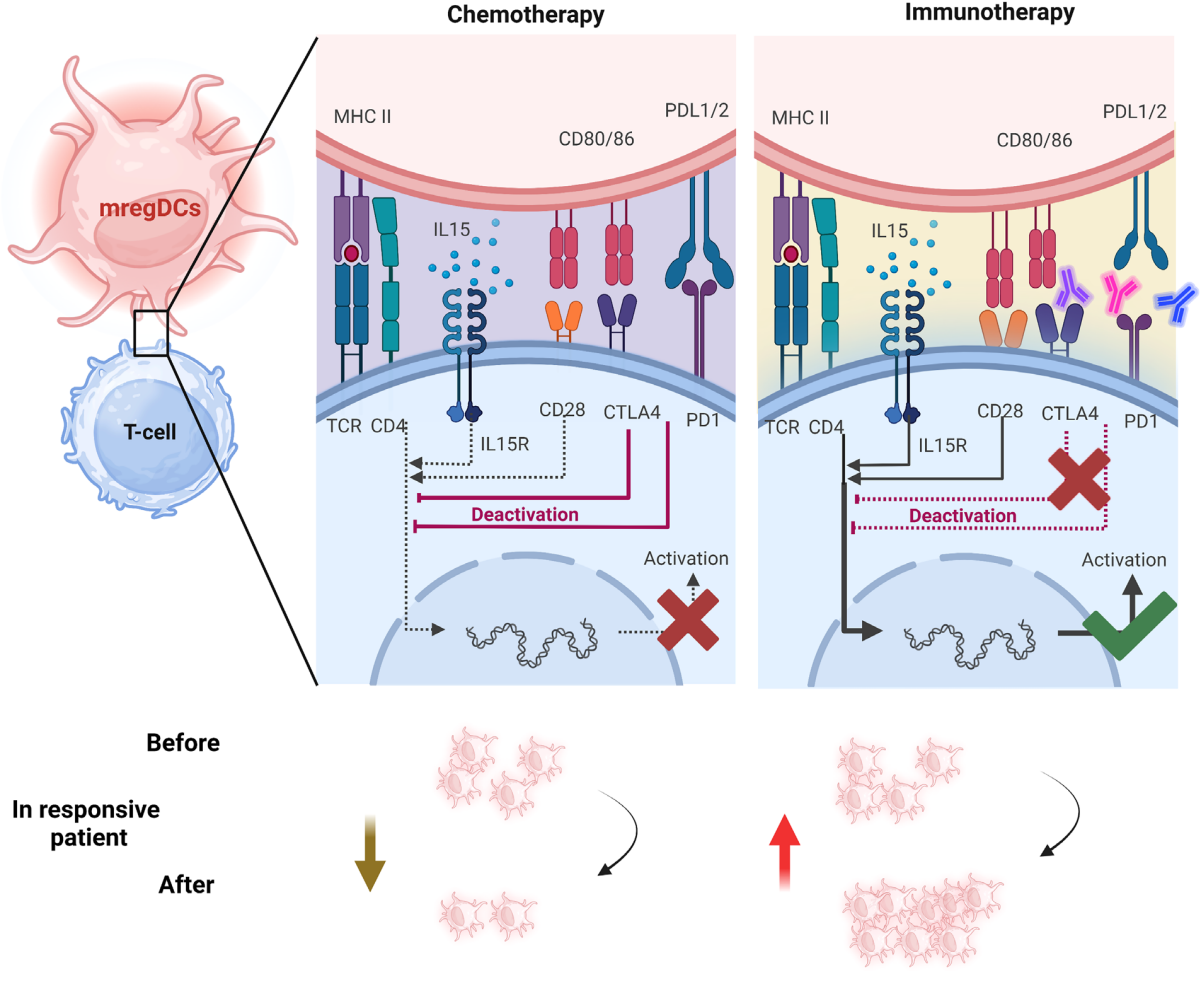
Mature dendritic cells enriched in immunoregulatory molecules (mregDCs) in chemotherapy and immunotherapy. In chemotherapy, immune costimulatory molecules (black line) and immune checkpoint ligands (red line) in mregDCs bind to their counterpart ligands on T cells. Due to the immunosuppressive microenvironment, the immune activation of mregDCs is much weaker and ultimately manifests as immunosuppression. While in immunotherapy, the suppressive immune checkpoints in mregDCs are blocked and therefore immune activation is enhanced. At the same time mregDCs are involved in the formation of tertiary lymph node structures, augmenting the efficacy of immunotherapy. During treatment, the number of mregDCs decreased in chemotherapy‐responsive patients and increased significantly in immunotherapy‐responsive patients. Created with BioRender.com.

As the most crucial and fundamental components of TLSs, B lymphocytes may be attracted to and activated by mregDCs via CCL19–CCR7 based on analysis of cell–cell interactions in lung adenocarcinoma.^30^ Marginal zone B cells have been demonstrated by Schriek et al.^62^ to extract MHC II from DCs to stimulate or influence T‐cell activation. In addition, a strong association between LAMP3^+^ DCs and high endothelial venules (HEVs) has been identified. Combination therapy with apatinib and an anti‐PD‐L1 triggers HEV production and promotes the infiltration of lymphocytes by activating LTβR signalling in mregDCs, thereby enhancing the antitumour immune response.^63^ Together, these findings robustly support the role of mregDCs in TLSs in cancer patients, indicating the function of mregDCs in coordinating adaptive anti‐immune responses at the tumour site (Figure 4).

## 6 mregDCs IN TUMOUR THERAPY

### 6.1 mregDCs in chemotherapy

To date, research on mregDCs in chemotherapy is relatively limited. Traditionally, the effects of chemotherapy on DCs are thought to be manifested in two ways.^64^ On the one hand, chemotherapy causes immunogenic death in tumour cells; it induces the recruitment and activation of DCs by generating various danger signals and damage‐associated molecular patterns. For instance, tumour‐derived IFN‐I is capable of recruiting and activating DCs, initiating the program of mregDCs and increasing their migratory and invasive capacity. Administration of ployI:C, a TLR3 agonist, resulted in activation of the STING pathway in mregDCs. Second, chemotherapy itself has an immunosuppressive effect, as it kills immune stores directly, leading to diminished effects on both local and systemic immunity. For instance, by releasing PGE2 from dying cancer cells, gemcitabine–cisplatin chemotherapy impedes DC maturation.^44^ Moreover, PGE2–EP2/EP4 signalling elicits an immunosuppressive microenvironment by potentiating the mregDC–Treg axis.^65^ Thus, upon pharmaceutical blockade of PGE2 release, synergism with chemotherapy could sensitise bladder tumours to anti‐PD‐1 ICB therapy. In colorectal cancer, mregDCs expressed LGALS9 and PD‐L1 (CD274) in tumour tissues from patients who received presurgical chemotherapy or not,^36^ while the production of chemokines recruiting Tregs, such as CCL19 and CCL10, was reduced in the treated patients. This phenomenon implies that chemotherapy modulates the phenotypes of mregDCs and attenuates the immunosuppressive effect of mregDCs.

### 6.2 mregDCs in immunotherapy

Evidence is increasing that mregDCs could serve as biomarkers of the response to immune checkpoint inhibition. As described above, one common feature of mregDCs in cancer is the overexpression of a wide spectrum of immunological checkpoints, for example, PD‐L1, TIM‐3, CD200 and IDO1. Consequently, it is feasible that mregDCs might be a potential target of ICB.

Recently, a large study of 499 triple‐negative breast cancer (TNBC) patients noted that PD‐L1 positivity was associated with significant increases in the infiltration of DCs, specifically mregDCs.^66^ Moreover, a recent study reported that the presence of PD‐L1 on myeloid cells was linked to an increased rate of pathologic complete response to anti‐PD‐1 treatment combined with chemotherapy.^67^ DCs, but just not macrophages, exhibited enhanced CD8^+^ T‐cell antitumour responses when PD‐L1 was deleted, significantly limiting tumour growth.^68^ This suggests that PD‐L1 expression in mregDCs, a vital target for immunology, is crucial in the response to ICB treatment.^69^ Evaluation of a gene expression dataset of pembrolizumab‐treated breast cancer patients revealed that the relative frequency of mregDCs correlated positively with T‐cell expansion following anti‐PD‐1 treatment, and mregDCs supported T‐cell function in responders, both at baseline and during treatment.^38^ Another study conducted on TNBC patients treated with paclitaxel monotherapy or paclitaxel plus atezolizumab in the neoadjuvant setting revealed a considerable increase in mregDCs in the combination treatment group versus the paclitaxel monotherapy group, indicating their involvement in the response to anti‐PD‐L1 therapy.^70^ Similar results have shown that the interaction between mregDCs and CD4^+^PD‐1^+^CXCL13^+^ T cells is critical for utilising the antitumour response caused by anti‐PD‐1 treatment.^71^ These results strongly indicate that PD‐L1 blockade reinvigorates mregDC function to generate a potent anticancer T‐cell immune response. Thus, a dependable way to estimate the number of mregDCs is considerably valuable for future immunotherapy research as it would allow the identification of patients predicted to have a poor immune response.

Preclinical research also suggests the potential of targeting inhibitory ligands of mregDCs to inhibit tumour progression. As mentioned, IDO is primarily released by mregDCs and inhibits CD8^+^ T‐cell proliferation and effector capabilities. Data from in vitro studies in melanoma demonstrate that targeted inhibition by the BTK–IDO checkpoint, in combination with PD‐L1 immune checkpoint inhibition, induces a synergistic therapeutic response.^72^

## 7 PROGNOSTIC VALUE OF mregDCs IN CANCERS

Given that mregDCs have been defined and studied mainly based on bioinformatics analysis of the single‐cell transcriptome, there is a paucity of information on their prognostic role in the clinic. An investigation of melanoma‐derived brain and meningeal metastases demonstrated that enrichment of mregDCs was linked to a more responsive immune environment and a higher overall survival (OS) rate in melanoma patients.^73^ Through the comprehensive analysis of multiple single‐cell datasets, Jaiswal et al.^74^ found that the features of mregDCs, the maturation of DCs and the coexpression of IFNγ are indicators of a favourable prognosis for melanoma patients.

Although the clinical implications of mregDC in cancers remain rudimentary, previous results emphasise the significant role that LAMP3, a typical marker of mregDC, plays in mediating better clinical outcomes of numerous solid cancers. The prognostic value of LAMP3^+^ DCs has been evaluated in several cancer types, including the ovarian cancer, melanoma, breast cancer and lung cancer. In an immunohistochemical study of metastatic melanoma, a high maximum density of LAMP3^+^ DCs was observed in primary tumours with activated T lymphocytes (*p* < .001) and was associated with prolonged OS (*p* = .0195).^75^ These findings were validated in another cohort of 458 patients; Elliott et al.^76^ reported that patients with a high density of LAMP3^+^ DCs (≥200/mm^2^) in sentinel lymph nodes containing metastatic melanoma cells had the lowest risk of death (*p* = .047).

In breast cancer, a recent report showed that recurrence‐free survival was better in patients with high mRNA levels of LAMP3 than in those with low levels in patients with basal or HER2‐positive subtypes.^77^ Notwithstanding, another study reported that the abundance of either mature DCs or infiltrating T cells did not significantly correlate with prognosis in other solid tumours, although there was a high correlation between the prevalence of LAMP3^+^ DCs and CD3^+^ cells and prognosis.^78^ In addition, high levels of activated cDC1s, which could be cDC1‐like mregDCs, were closely linked with better survival in patients diagnosed with colorectal cancer.^11^ Another study used qPCR (*n* = 32) and immunohistochemistry (*n* = 192) to assess colorectal carcinoma and found that patients with relatively high densities of CD8^+^ T cells and LAMP3^+^ DCs had longer OS than those with low densities (*p* = .008).^79^ In lung cancer, a study using immunohistochemistry staining found that patients who had considerably higher concentrations of CD8^+^ T cells and LAMP3^+^ DCs than those who had lower densities had prolonged OS in early stage.^55^ An investigation with independent retrospective cohorts of ovarian carcinoma patients revealed that robust tumour infiltration by LAMP3^+^ DCs was associated with Th1 polarisation, cytotoxic activity as well as improved OS.^80^ Similarly, tumour‐infiltrating LAMP3^+^DCs were commonly prevalent in the peritumoural region and accompanied by CD8^+^ T cells, and high levels of these DCs were highly correlated with a good prognosis among patients with ESCC.^81^ In HNSCC, Hoffman et al.^71^ found that patients with an enriched cDC2‐like mregDCs phenotype had a better prognosis than other patients and had a favourable immunotherapy response. A similar phenomenon was also demonstrated in breast cancer and melanoma patients.

These results highlight the predictive value of mregDCs in cancers, and evidence indicates that mregDCs are associated with improved clinical outcomes. Although several preclinical studies have suggested an immunosuppressive function of mregDCs within the TME, reports are still lacking relating tumour‐infiltrating mregDCs with poor prognosis and immunotherapy resistance in cancer patients.^82^ This phenomenon may be due to the lack of a unified definition standard for mregDCs, which has resulted in multiple and complex names in different studies; these names include LAMP3^+^CCR7^+^ cells, CD45^+^Lin^−^MHC‐II^+^CD11c^+^CD80^hi^CD274^hi^, and LAMP3^+^IDO1^+^CCL19^+^ cells. Furthermore, the annotation of mregDCs in single‐cell studies involves a series of genes, while previous relevant studies could not precisely define mregDCs due to the limitations of the experimental technology. Therefore, the findings of immunohistochemistry or flow cytometric assays may not perfectly match the results of single‐cell transcriptome studies. Finally, the dual immunomodulatory function of mregDCs may be altered during different treatments, resulting in inconsistent results regarding their prognostic value. For reliable prognosis evaluation in cancers, better markers and a unified definition of mregDCs are urgently needed. Additionally, further studies with more advanced techniques are needed to substantiate the prognostic value of mregDCs.

## 8 POTENTIAL CLINICAL IMPLICATIONS

**FIGURE 5:**
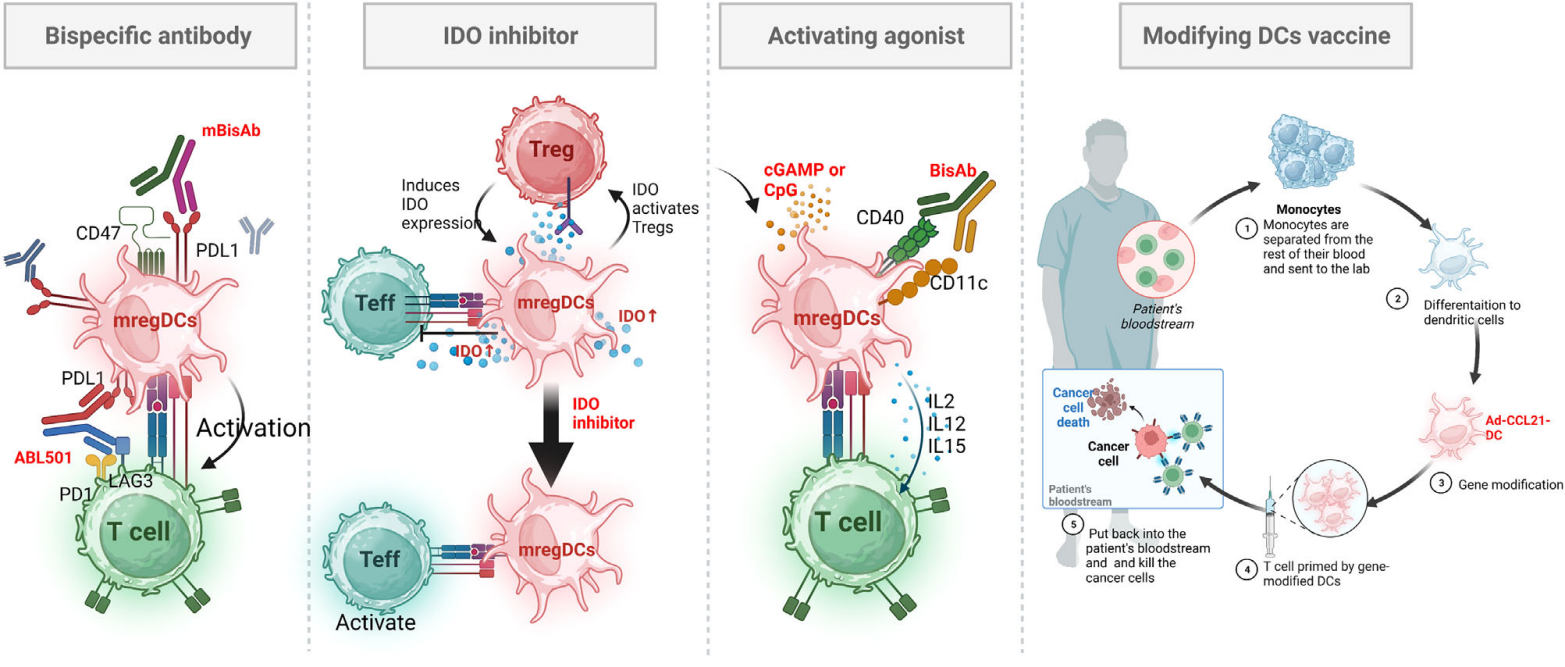
Immunotherapeutic strategies for targeting mature dendritic cells enriched in immunoregulatory molecules (mregDCs). Strategy for targeting mregDCs to achieve antitumour effects. (1) mregDCs highly express immune checkpoint ligands, and most mregDCs can be targeted using specific inhibitor antibodies (red) to block immunosuppressive switches, such as ABL501. (2) Blocking immunosuppressive factors (such as IDO inhibitors) in the microenvironment activates pro‐inflammatory pathways (cyclic guanosine monophosphate‐adenosine monophosphate (cGAMP) or cytosine‐phosphate‐guanine (CpG)) in mregDCs and can re‐educate mregDCs to support antitumour immunity. (3) Use of agonists that activate dendritic cell activation can help enhance the antitumour function of mregDCs and prompt T‐cell initiation and differentiation. (4) In addition, reprogramming the genes of powerful antitumour functions in mregDCs enables DCs to acquire more potent antitumour functions and prolong the survival of patients. Created with BioRender.com.

Considering the high level of immune checkpoint ligands on mregDCs, simultaneous targeting of immune checkpoint ligands on mregDCs and immune checkpoint receptors on T cells may yield synergistic effects, resulting in robust antitumour immunity. Several preclinical studies have demonstrated that combination with other immune checkpoint inhibitors may significantly improve the antitumour effect of anti‐PD‐1/PD‐L1 therapies (Figure 5).

The relevance of TIM‐3 to the mregDC program was recently discovered by a study employing a genetic knockout mouse model and scRNA‐seq. This study indicated that deletion of TIM‐3 on DCs prevented the acquisition of the mregDC program, which promotes the preservation of stem‐like T cells and CD8^+^ effector cells. Moreover, when anti‐TIM‐3 and anti‐PD‐L1 treatments were combined, the tumour burden was significantly reduced.^37^ Furthermore, TIM‐3 inhibition was found to cause cDC1‐like mregDCs to take up more tumour antigen and activate the cGAS–STING pathway,^83^ inducing the release of CXCL9 and IL‐12 from mregDCs and encouraging CD8^+^ T‐cell colocalisation with DCs to enhance antitumour immunity.^84^ The bispecific antibody ABL501, which targets both LAG‐3 and PD‐L1, promotes mregDC interaction with T cells and consequently induces efficient CD8^+^ T‐cell responses.^85^ More importantly, blocking the mregDC and Treg interaction could promote their communication with effector T cells. Targeting mregDCs and PD‐1^+^ T cells yielded a synergistic effect in prostate cancer.^86^ Blocking CD47 and PD‐L1 by bispecific antibody could increase the antigen processing and presentation function of mregDCs and increase the frequency of stem‐like progenitor and effector CD8^+^ T‐cell subsets in the tumour, achieving an effective response to ICB therapy.^87^ Collectively, these results imply that synergistic targeting of the immune checkpoint molecules of T cells and mregDCs exhibits significant therapeutic utility.

In addition to targeting mregDC immune checkpoint‐related molecules, therapeutics targeting mregDCs in cancer can take two approaches, the first of which is augmenting the mregDCs activation state to prime T cells. A bispecific antibody created by Dahan and coworkers^88^ that targets CD40 and CD11c widens the therapeutic window of CD40 agonists via specific DC expansion and activation, triggering more mregDCs to effectively prime T cells. Moreover, Fc‐enhanced CD40 antibody agonists targeting DCs exhibit robust and long‐lasting systemic antitumour immunity.^89^ By promoting CD8^+^ T‐cell infiltration and local TLS neogenesis, STING agonists also assist mregDCs control tumour development.^90^ Notably, STING‐type I IFN from microbiota enhances the responsiveness to ICB in melanoma patients by enhancing DC–NK cell crosstalk.^91^ The second approach is modifying DC cancer vaccines. Although the quantity of mregDCs increases in response to ICB treatment, they still remain a moderately small percentage of antigen‐introducing cells. The restricted amount of activated DCs is likely to result in unsatisfactory antigen cross‐presentation for clinical needs. As mentioned earlier, migration of CCR7^+^ mregDCs and efficient interactions between T cells and DCs are mediated by CCL21. In a phase I trial (NCT01574222), patients with lung cancer who received injections of CCL21‐modified DCs exhibited tumour‐specific immune responses. PD‐L1 expression increased in tandem with increasing CD8^+^ T‐cell infiltration.^92^ Therefore, as a result, patients who lack CD8^+^ T infiltrate and have low PD‐L1 expression have extraordinary potential for combination therapy that combines anti‐PD‐1/PD‐L1 medicines with modified DC vaccines. Considering the high expression of SOCS1 in mregDCs, genetically modified DCs with SOCS1 silencing were evaluated (NCT01956630) and were found to elicit powerful immune effects and increase the survival of patients with acute myeloid leukaemia.^93^ Furthermore, the substantially conserved properties of mregDCs observed across various cancers support the hypothesis that focusing shared immunotherapy paradigms on mregDCs could help a great number of cancer patients, irrespective of disease heterogeneity.^27^

## 9 CONCLUSIONS AND PERSPECTIVES

mregDCs research has recently gained considerable momentum. It is known that mregDCs represent DCs with distinct programs conserved across lineages and play dynamic functions in the TME, either promoting cancer elimination or immune suppression via direct or indirect mechanisms, but the details are unclear. In addition, infiltration of LAMP3^+^ mregDCs is positively correlated with favourable prognosis in multiple cancer types, while the prognostic value of other subsets of mregDCs remains undetermined and will require further investigation. Remarkably, preclinical studies and early‐stage clinical trials indicate that mregDCs play critical roles in ICB treatment, and targeting mregDCs by augmenting their immunostimulatory function or weakening their immunosuppressive role presents tremendous opportunities as a promising therapeutic approach. Collectively, accumulating results have identified mregDCs as a novel and important population of DCs in cancers, and further research is required to better understand the molecular system that dynamically regulates their development and maintenance in the TME; such studies will provide deep insights to increase the functionality of this DC population.

## CONFLICT OF INTEREST STATEMENT

The authors declare no conflicts of interest.

## References

[1] Guilliams M , Ginhoux F , Jakubzick C , et al. Dendritic cells, monocytes and macrophages: a unified nomenclature based on ontogeny. Nat Rev Immunol. 2014;14(8):571‐578. 25033907 10.1038/nri3712 PMC4638219

[2] Cabeza‐Cabrerizo M , Cardoso A , Minutti CM , Pereira da Costa M , Reis e Sousa C . Dendritic cells revisited. Annu Rev Immunol. 2021;39:131‐166. 33481643 10.1146/annurev-immunol-061020-053707

[3] Schetters STT , Rodriguez E , Kruijssen LJW , et al. Monocyte‐derived APCs are central to the response of PD1 checkpoint blockade and provide a therapeutic target for combination therapy. J Immunother Cancer. 2020;8(2):e000588. 32690667 10.1136/jitc-2020-000588 PMC7371367

[4] Villani AC , Satija R , Reynolds G , et al. Single‐cell RNA‐seq reveals new types of human blood dendritic cells, monocytes, and progenitors. Science. 2017;356(6335):eaah4573. 10.1126/science.aah4573 PMC5775029 28428369

[5] Bourdely P , Anselmi G , Vaivode K , et al. Transcriptional and functional analysis of CD1c(+) human dendritic cells identifies a CD163(+) subset priming CD8(+)CD103(+) T cells. Immunity. 2020;53(2):335‐352.e338. 32610077 10.1016/j.immuni.2020.06.002 PMC7445430

[6] Maier B , Leader AM , Chen ST , et al. A conserved dendritic‐cell regulatory program limits antitumour immunity. Nature. 2020;580(7802):257‐262. 32269339 10.1038/s41586-020-2134-y PMC7787191

[7] Zilionis R , Engblom C , Pfirschke C , et al. Single‐cell transcriptomics of human and mouse lung cancers reveals conserved myeloid populations across individuals and species. Immunity. 2019;50(5):1317‐1334.e1310. 30979687 10.1016/j.immuni.2019.03.009 PMC6620049

[8] Zhang Q , He Y , Luo N , et al. Landscape and dynamics of single immune cells in hepatocellular carcinoma. Cell. 2019;179(4):829‐845.e820. 31675496 10.1016/j.cell.2019.10.003

[9] Ginhoux F , Guilliams M , Merad M . Expanding dendritic cell nomenclature in the single‐cell era. Nat Rev Immunol. 2022;22(2):67‐68. 35027741 10.1038/s41577-022-00675-7

[10] Sun Y , Wu L , Zhong Y , et al. Single‐cell landscape of the ecosystem in early‐relapse hepatocellular carcinoma. Cell. 2021;184(2):404‐421.e416. 33357445 10.1016/j.cell.2020.11.041

[11] Zhang L , Li Z , Skrzypczynska KM , et al. Single‐cell analyses inform mechanisms of myeloid‐targeted therapies in colon cancer. Cell. 2020;181(2):442‐459.e429. 32302573 10.1016/j.cell.2020.03.048

[12] Nirmal AJ , Maliga Z , Vallius T , et al. The spatial landscape of progression and immunoediting in primary melanoma at single cell resolution. Cancer Discov. 2022;;12(6):1518‐1541. 35404441 10.1158/2159-8290.CD-21-1357 PMC9167783

[13] Kürten CHL , Kulkarni A , Cillo AR , et al. Investigating immune and non‐immune cell interactions in head and neck tumors by single‐cell RNA sequencing. Nat Commun. 2021;12(1):7338. 34921143 10.1038/s41467-021-27619-4 PMC8683505

[14] Wu SZ , Al‐Eryani G , Roden DL , et al. A single‐cell and spatially resolved atlas of human breast cancers. Nat Genet. 2021;53(9):1334‐1347. 34493872 10.1038/s41588-021-00911-1 PMC9044823

[15] Zheng Y , Chen Z , Han Y , et al. Immune suppressive landscape in the human esophageal squamous cell carcinoma microenvironment. Nat Commun. 2020;11(1):6268. 33293583 10.1038/s41467-020-20019-0 PMC7722722

[16] Liu Y , He S , Wang X‐L , et al. Tumour heterogeneity and intercellular networks of nasopharyngeal carcinoma at single cell resolution. Nat Commun. 2021;12(1):741. 33531485 10.1038/s41467-021-21043-4 PMC7854640

[17] Zhang X , Peng L , Luo Y , et al. Dissecting esophageal squamous‐cell carcinoma ecosystem by single‐cell transcriptomic analysis. Nat Commun. 2021;12(1):5291. 34489433 10.1038/s41467-021-25539-x PMC8421382

[18] Wu R , Ohara RA , Jo S , et al. Mechanisms of CD40‐dependent cDC1 licensing beyond costimulation. Nat Immunol. 2022;23(11):1536‐1550. 36271147 10.1038/s41590-022-01324-w PMC9896965

[19] Alessandrini F , Pezzè L , Ciribilli Y . LAMPs: shedding light on cancer biology. Semin Oncol. 2017;44(4):239‐253. 29526252 10.1053/j.seminoncol.2017.10.013

[20] Qian J , Olbrecht S , Boeckx B , et al. A pan‐cancer blueprint of the heterogeneous tumor microenvironment revealed by single‐cell profiling. Cell Res. 2020;30(9):745‐762. 32561858 10.1038/s41422-020-0355-0 PMC7608385

[21] Movassagh M , Spatz A , Davoust J , et al. Selective accumulation of mature DC‐Lamp+ dendritic cells in tumor sites is associated with efficient T‐cell‐mediated antitumor response and control of metastatic dissemination in melanoma. Cancer Res. 2004;64(6):2192‐2198. 15026362 10.1158/0008-5472.can-03-2969

[22] Wu H , Qin J , Zhao Q , Lu L , Li C . Microdissection of the bulk transcriptome at single‐cell resolution reveals clinical significance and myeloid cells heterogeneity in lung adenocarcinoma. Front Immunol. 2021;12:723908. 34659209 10.3389/fimmu.2021.723908 PMC8515901

[23] Bembenek A , Li J , Loddenkemper C , et al. Presence of mature DC‐Lamp+ dendritic cells in sentinel and non‐sentinel lymph nodes of breast cancer patients. Eur J Surg Oncol. 2008;34(5):514‐518. 17618075 10.1016/j.ejso.2007.05.013

[24] Vaahtomeri K , Brown M , Hauschild R , et al. Locally triggered release of the chemokine CCL21 promotes dendritic cell transmigration across lymphatic endothelia. Cell Rep. 2017;19(5):902‐909. 28467903 10.1016/j.celrep.2017.04.027 PMC5437727

[25] Liu J , Zhang X , Chen K , et al. CCR7 chemokine receptor‐inducible lnc‐Dpf3 restrains dendritic cell migration by inhibiting HIF‐1α‐mediated glycolysis. Immunity. 2019;50(3):600‐615.e615. 30824325 10.1016/j.immuni.2019.01.021

[26] Chen Z , Zhou L , Liu L , et al. Single‐cell RNA sequencing highlights the role of inflammatory cancer‐associated fibroblasts in bladder urothelial carcinoma. Nat Commun. 2020;11(1):5077. 33033240 10.1038/s41467-020-18916-5 PMC7545162

[27] Leader AM , Grout JA , Maier BB , et al. Single‐cell analysis of human non‐small cell lung cancer lesions refines tumor classification and patient stratification. Cancer Cell. 2021;39(12):1594‐1609.e1512. 34767762 10.1016/j.ccell.2021.10.009 PMC8728963

[28] Mair F , Erickson JR , Frutoso M , et al. Extricating human tumour immune alterations from tissue inflammation. Nature. 2022;605(7911):728‐735. 35545675 10.1038/s41586-022-04718-w PMC9132772

[29] Lee AH , Sun L , Mochizuki AY , et al. Neoadjuvant PD‐1 blockade induces T cell and cDC1 activation but fails to overcome the immunosuppressive tumor associated macrophages in recurrent glioblastoma. Nat Commun. 2021;12(1):6938. 34836966 10.1038/s41467-021-26940-2 PMC8626557

[30] Yang L , He Y‐T , Dong S , et al. Single‐cell transcriptome analysis revealed a suppressive tumor immune microenvironment in EGFR mutant lung adenocarcinoma. J Immunother Cancer. 2022;10(2):e003534. 35140113 10.1136/jitc-2021-003534 PMC8830346

[31] Wang S , Wu Q , Chen T , et al. Blocking CD47 promotes anti‐tumor immunity through CD103+ dendritic cell‐NK cell axis in murine hepatocellular carcinoma model. J Hepatol. 2022;77(2):467‐478. 35367532 10.1016/j.jhep.2022.03.011

[32] Di Pilato M , Kfuri‐Rubens R , Pruessmann JN , et al. CXCR6 positions cytotoxic T cells to receive critical survival signals in the tumor microenvironment. Cell. 2021;184(17):4512‐4530.e4522. 34343496 10.1016/j.cell.2021.07.015 PMC8719451

[33] Dieu‐Nosjean MC , Antoine M , Danel C , et al. Long‐term survival for patients with non‐small‐cell lung cancer with intratumoral lymphoid structures. J Clin Oncol. 2008;26(27):4410‐4417. 18802153 10.1200/JCO.2007.15.0284

[34] Cheng S , Li Z , Gao R , et al. A pan‐cancer single‐cell transcriptional atlas of tumor infiltrating myeloid cells. Cell. 2021;184(3):792‐809.e723. 33545035 10.1016/j.cell.2021.01.010

[35] Peng WS , Zhou X , Yan W‐B , et al. Dissecting the heterogeneity of the microenvironment in primary and recurrent nasopharyngeal carcinomas using single‐cell RNA sequencing. OncoImmunology. 2022;11(1):2026583. 35096485 10.1080/2162402X.2022.2026583 PMC8794254

[36] Che LH , Liu J‐W , Huo J‐P , et al. A single‐cell atlas of liver metastases of colorectal cancer reveals reprogramming of the tumor microenvironment in response to preoperative chemotherapy. Cell Discov. 2021;7(1):80. 34489408 10.1038/s41421-021-00312-y PMC8421363

[37] Dixon KO , Tabaka M , Schramm MA , et al. TIM‐3 restrains anti‐tumour immunity by regulating inflammasome activation. Nature. 2021;595(7865):101‐106. 34108686 10.1038/s41586-021-03626-9 PMC8627694

[38] Bassez A , Vos H , Dyck LV , et al. A single‐cell map of intratumoral changes during anti‐PD1 treatment of patients with breast cancer. Nat Med. 2021;27(5):820‐832. 33958794 10.1038/s41591-021-01323-8

[39] Ardouin L , Luche H , Chelbi R , et al. Broad and largely concordant molecular changes characterize tolerogenic and immunogenic dendritic cell maturation in thymus and periphery. Immunity. 2016;45(2):305‐318. 27533013 10.1016/j.immuni.2016.07.019

[40] Tang‐Huau TL , Gueguen P , Goudot C , et al. Human in vivo‐generated monocyte‐derived dendritic cells and macrophages cross‐present antigens through a vacuolar pathway. Nat Commun. 2018;9(1):2570. 29967419 10.1038/s41467-018-04985-0 PMC6028641

[41] Chen YL , Gomes T , Hardman CS , et al. Re‐evaluation of human BDCA‐2+ DC during acute sterile skin inflammation. J Exp Med. 2020;217(3). 10.1084/jem.20190811 PMC7062525 31845972

[42] Manh TP , Alexandre Y , Baranek T , Crozat K , Dalod M . Plasmacytoid, conventional, and monocyte‐derived dendritic cells undergo a profound and convergent genetic reprogramming during their maturation. Eur J Immunol. 2013;43(7):1706‐1715. 23553052 10.1002/eji.201243106 PMC3799015

[43] Baldominos P , Barbera‐Mourelle A , Barreiro O , et al. Quiescent cancer cells resist T cell attack by forming an immunosuppressive niche. Cell. 2022;185(10):1694‐1708.e1619. 35447074 10.1016/j.cell.2022.03.033 PMC11332067

[44] Nikolos F , Hayashi K , Hoi XP , et al. Cell death‐induced immunogenicity enhances chemoimmunotherapeutic response by converting immune‐excluded into T‐cell inflamed bladder tumors. Nat Commun. 2022;13(1):1487. 35347124 10.1038/s41467-022-29026-9 PMC8960844

[45] Li Y , Jeong J , Song W . Molecular characteristics and distribution of adult human corneal immune cell types. Front Immunol. 2022;13:798346. 35280984 10.3389/fimmu.2022.798346 PMC8905655

[46] Nakamizo S , Dutertre C‐A , Khalilnezhad A , et al. Single‐cell analysis of human skin identifies CD14+ type 3 dendritic cells co‐producing IL1B and IL23A in psoriasis. J Exp Med. 2021;218(9):e20202345. 10.1084/jem.20202345 PMC8292131 34279540

[47] Park JE , Botting RA , Conde CD , et al. A cell atlas of human thymic development defines T cell repertoire formation. Science. 2020;367(6480):eaay3224. 10.1126/science.aay3224 PMC7611066 32079746

[48] He H , Suryawanshi H , Morozov P , et al. Single‐cell transcriptome analysis of human skin identifies novel fibroblast subpopulation and enrichment of immune subsets in atopic dermatitis. J Allergy Clin Immunol. 2020;145(6):1615‐1628. 32035984 10.1016/j.jaci.2020.01.042

[49] Yao RQ , Li Z‐x , Wang L‐x , et al. Single‐cell transcriptome profiling of the immune space‐time landscape reveals dendritic cell regulatory program in polymicrobial sepsis. Theranostics. 2022;12(10):4606‐4628. 35832091 10.7150/thno.72760 PMC9254255

[50] Rindler K , Bauer WM , Jonak C , et al. Single‐cell RNA sequencing reveals tissue compartment‐specific plasticity of mycosis fungoides tumor cells. Front Immunol. 2021;12:666935. 33968070 10.3389/fimmu.2021.666935 PMC8097053

[51] Binnewies M , Mujal AM , Pollack JL , et al. Unleashing type‐2 dendritic cells to drive protective antitumor CD4(+) T cell immunity. Cell. 2019;177(3):556‐571.e516. 30955881 10.1016/j.cell.2019.02.005 PMC6954108

[52] Kersten K , Hu KH , Combes AJ , et al. Spatiotemporal co‐dependency between macrophages and exhausted CD8(+) T cells in cancer. Cancer Cell. 2022;40(6):624‐638.e629. 35623342 10.1016/j.ccell.2022.05.004 PMC9197962

[53] Marshall LA , Marubayashi S , Jorapur A , et al. Tumors establish resistance to immunotherapy by regulating T(reg) recruitment via CCR4. J Immunother Cancer. 2020;8(2):e000764. 33243932 10.1136/jitc-2020-000764 PMC7692993

[54] Gargaro M , Scalisi G , Manni G , et al. Indoleamine 2,3‐dioxygenase 1 activation in mature cDC1 promotes tolerogenic education of inflammatory cDC2 via metabolic communication. Immunity. 2022;55(6):1032‐1050.e1014. 35704993 10.1016/j.immuni.2022.05.013 PMC9220322

[55] Germain C , Gnjatic S , Tamzalit F , et al. Presence of B cells in tertiary lymphoid structures is associated with a protective immunity in patients with lung cancer. Am J Respir Crit Care Med. 2014;189(7):832‐844. 24484236 10.1164/rccm.201309-1611OC

[56] Bell D , Chomarat P , Broyles D , et al. In breast carcinoma tissue, immature dendritic cells reside within the tumor, whereas mature dendritic cells are located in peritumoral areas. J Exp Med. 1999;190(10):1417‐1426. 10562317 10.1084/jem.190.10.1417 PMC2195690

[57] Liu J , Lu G , Li Z , et al. Distinct compartmental distribution of mature and immature dendritic cells in esophageal squamous cell carcinoma. Pathol Res Pract. 2010;206(9):602‐606. 20547010 10.1016/j.prp.2010.03.011

[58] Horeweg N , Workel H , Loiero D , et al. Tertiary lymphoid structures critical for prognosis in endometrial cancer patients. Nat Commun. 2022;13(1):1373. 35296668 10.1038/s41467-022-29040-x PMC8927106

[59] Wang B , Liu J , Han Y , Deng Y , Li J , Jiang Y , et al. The presence of tertiary lymphoid structures provides new insight into the clinicopathological features and prognosis of patients with breast cancer. Front Immunol. 2022;13:868155. 35664009 10.3389/fimmu.2022.868155 PMC9161084

[60] Abdulrahman Z , Santegoets SJ , Sturm G , et al. Tumor‐specific T cells support chemokine‐driven spatial organization of intratumoral immune microaggregates needed for long survival. J Immunother Cancer. 2022;10(2):e004346. 35217577 10.1136/jitc-2021-004346 PMC8883276

[61] Cohen M , Giladi A , Barboy O , et al. The interaction of CD4(+) helper T cells with dendritic cells shapes the tumor microenvironment and immune checkpoint blockade response. Nat Cancer. 2022;3(3):303‐317. 35241835 10.1038/s43018-022-00338-5

[62] Schriek P , Ching AC , Moily NS , et al. Marginal zone B cells acquire dendritic cell functions by trogocytosis. Science. 2022;375(6581):eabf7470. 35143312 10.1126/science.abf7470

[63] Zhang Y , Wang F , Sun H‐r , et al. Apatinib combined with PD‐L1 blockade synergistically enhances antitumor immune responses and promotes HEV formation in gastric cancer. J Cancer Res Clin Oncol. 2021;147(8):2209‐2222. 33891173 10.1007/s00432-021-03633-3 PMC11802163

[64] Hayashi K , Nikolos F , Lee YC , et al. Tipping the immunostimulatory and inhibitory DAMP balance to harness immunogenic cell death. Nat Commun. 2020;11(1):6299. 33288764 10.1038/s41467-020-19970-9 PMC7721802

[65] Thumkeo D , Punyawatthananukool S , Prasongtanakij S , et al. PGE(2)‐EP2/EP4 signaling elicits immunosuppression by driving the mregDC‐Treg axis in inflammatory tumor microenvironment. Cell Rep. 2022;39(10):110914. 35675777 10.1016/j.celrep.2022.110914

[66] Carter JM , Polley M‐YC , Leon‐Ferre RA , et al. Characteristics and spatially defined immune (micro)landscapes of early‐stage PD‐L1‐positive triple‐negative breast cancer. Clin Cancer Res. 2021;27(20):5628‐5637. 34108182 10.1158/1078-0432.CCR-21-0343 PMC8808363

[67] Ahmed FS , Gaule P , McGuire J , et al. PD‐L1 protein expression on both tumor cells and macrophages are associated with response to neoadjuvant durvalumab with chemotherapy in triple‐negative breast cancer. Clin Cancer Res. 2020;26(20):5456‐5461. 32709714 10.1158/1078-0432.CCR-20-1303 PMC7572612

[68] Xia T , Li K , Niu N , et al. Immune cell atlas of cholangiocarcinomas reveals distinct tumor microenvironments and associated prognoses. J Hematol Oncol. 2022;15(1):37. 35346322 10.1186/s13045-022-01253-z PMC8962046

[69] Peng Q , Qiu X , Zhang Z , et al. PD‐L1 on dendritic cells attenuates T cell activation and regulates response to immune checkpoint blockade. Nat Commun. 2020;11(1):4835. 32973173 10.1038/s41467-020-18570-x PMC7518441

[70] Zhang Y , Chen H , Mo H , et al. Single‐cell analyses reveal key immune cell subsets associated with response to PD‐L1 blockade in triple‐negative breast cancer. Cancer Cell. 2021;39(12):1578‐1593.e1578. 34653365 10.1016/j.ccell.2021.09.010

[71] Hoffmann C , Noel F , Grandclaudon M , et al. PD‐L1 and ICOSL discriminate human secretory and helper dendritic cells in cancer, allergy and autoimmunity. Nat Commun. 2022;13(1):1983. 35418195 10.1038/s41467-022-29516-w PMC9008048

[72] Sharma MD , Pacholczyk R , Shi H , et al. Inhibition of the BTK‐IDO‐mTOR axis promotes differentiation of monocyte‐lineage dendritic cells and enhances anti‐tumor T cell immunity. IMMUNITY. 2021;54(10):2354‐2371.e2358. 34614413 10.1016/j.immuni.2021.09.005 PMC8516719

[73] Smalley I , Chen Z , Phadke M , et al. Single‐cell characterization of the immune microenvironment of melanoma brain and leptomeningeal metastases. Clin Cancer Res. 2021;27(14):4109‐4125. 34035069 10.1158/1078-0432.CCR-21-1694 PMC8282775

[74] Jaiswal A , Verma A , Dannenfelser R , et al. An activation to memory differentiation trajectory of tumor‐infiltrating lymphocytes informs metastatic melanoma outcomes. Cancer Cell. 2022;40(5):524‐544.e525. 35537413 10.1016/j.ccell.2022.04.005 PMC9122099

[75] Ladányi A , Kiss J , Somlai B , et al. Density of DC‐LAMP(+) mature dendritic cells in combination with activated T lymphocytes infiltrating primary cutaneous melanoma is a strong independent prognostic factor. Cancer Immunol Immunother. 2007;56(9):1459‐1469. 17279413 10.1007/s00262-007-0286-3 PMC11030123

[76] Elliott B , Scolyer RA , Suciu S , et al. Long‐term protective effect of mature DC‐LAMP+ dendritic cell accumulation in sentinel lymph nodes containing micrometastatic melanoma. Clin Cancer Res. 2007;13(13):3825‐3830. 17606713 10.1158/1078-0432.CCR-07-0358

[77] de Mingo Pulido Á , Gardner A , Hiebler S , et al. TIM‐3 regulates CD103(+) dendritic cell function and response to chemotherapy in breast cancer. Cancer Cell. 2018;33(1):60‐74.e66. 29316433 10.1016/j.ccell.2017.11.019 PMC5764109

[78] Treilleux I , Blay J‐Y , Bendriss‐Vermare N , et al. Dendritic cell infiltration and prognosis of early stage breast cancer. Clin Cancer Res. 2004;10(22):7466‐7474. 15569976 10.1158/1078-0432.CCR-04-0684

[79] Remark R , Alifano M , Cremer I , et al. Characteristics and clinical impacts of the immune environments in colorectal and renal cell carcinoma lung metastases: influence of tumor origin. Clin Cancer Res. 2013;19(15):4079‐4091. 23785047 10.1158/1078-0432.CCR-12-3847

[80] Truxova I , Kasikova L , Hensler M , et al. Mature dendritic cells correlate with favorable immune infiltrate and improved prognosis in ovarian carcinoma patients. J Immunother Cancer. 2018;6(1):139. 30526667 10.1186/s40425-018-0446-3 PMC6288908

[81] Nishimura J , Tanaka H , Yamakoshi Y , et al. Impact of tumor‐infiltrating LAMP‐3 dendritic cells on the prognosis of esophageal squamous cell carcinoma. Esophagus. 2019;16(4):333‐344. 30968254 10.1007/s10388-019-00669-w

[82] Gupta YH , Khanom A , Acton SE . Control of dendritic cell function within the tumour microenvironment. Front Immunol. 2022;13:733800. 35355992 10.3389/fimmu.2022.733800 PMC8960065

[83] de Mingo Pulido Á , Hänggi K , Celias DP , et al. The inhibitory receptor TIM‐3 limits activation of the cGAS‐STING pathway in intra‐tumoral dendritic cells by suppressing extracellular DNA uptake. Immunity. 2021;54(6):1154‐1167.e1157. 33979578 10.1016/j.immuni.2021.04.019 PMC8192496

[84] Gardner A , de Mingo Pulido A , Hänggi K , et al. TIM‐3 blockade enhances IL‐12‐dependent antitumor immunity by promoting CD8(+) T cell and XCR1(+) dendritic cell spatial co‐localization. J Immunother Cancer. 2022;10(1):e003571. 10.1136/jitc-2021-003571 PMC8734033 34987021

[85] Sung E , Ko M , Won J‐Y , et al. LAG‐3xPD‐L1 bispecific antibody potentiates antitumor responses of T cells through dendritic cell activation. Mol Ther. 2022;30(8):2800‐2816. 10.1016/j.ymthe.2022.05.003 PMC9372323 35526096

[86] Lu X , Horner JW , Paul E , et al. Effective combinatorial immunotherapy for castration‐resistant prostate cancer. Nature. 2017;543(7647):728‐732. 28321130 10.1038/nature21676 PMC5374023

[87] Chen SH , Dominik PK , Stanfield J , et al. Dual checkpoint blockade of CD47 and PD‐L1 using an affinity‐tuned bispecific antibody maximizes antitumor immunity. J Immunother Cancer. 2021;9(10):e003464. 34599020 10.1136/jitc-2021-003464 PMC8488710

[88] Salomon R , Rotem H , Katzenelenbogen Y , et al. Bispecific antibodies increase the therapeutic window of CD40 agonists through selective dendritic cell targeting. Nat Cancer. 2022;3(3):287‐302. 35190724 10.1038/s43018-022-00329-6

[89] Garris CS , Wong JL , Ravetch JV , Knorr DA . Dendritic cell targeting with Fc‐enhanced CD40 antibody agonists induces durable antitumor immunity in humanized mouse models of bladder cancer. Sci Transl Med. 2021;13(594):eabd1346. 10.1126/scitranslmed.abd1346 PMC8325152 34011627

[90] Chelvanambi M , Fecek RJ , Taylor JL , Storkus WJ . STING agonist‐based treatment promotes vascular normalization and tertiary lymphoid structure formation in the therapeutic melanoma microenvironment. J Immunother Cancer. 2021;9(2):e001906. 10.1136/jitc-2020-001906 PMC7852948 33526609

[91] Lam KC , Araya RE , Huang A , et al. Microbiota triggers STING‐type I IFN‐dependent monocyte reprogramming of the tumor microenvironment. Cell. 2021;184(21):5338‐5356.e5321. 34624222 10.1016/j.cell.2021.09.019 PMC8650838

[92] Lee JM , Lee M‐H , Garon E , et al. Phase I trial of intratumoral injection of CCL21 gene‐modified dendritic cells in lung cancer elicits tumor‐specific immune responses and CD8(+) T‐cell infiltration. Clin Cancer Res. 2017;23(16):4556‐4568. 28468947 10.1158/1078-0432.CCR-16-2821 PMC5599263

[93] Wang D , Huang XF , Hong B , et al. Efficacy of intracellular immune checkpoint‐silenced DC vaccine. JCI Insight. 2018;3(3):e98368. 10.1172/jci.insight.98368 PMC5821183 29415891

[94] Sinjab A , Han G , Treekitkarnmongkol W , et al. Resolving the spatial and cellular architecture of lung adenocarcinoma by multiregion single‐cell sequencing. Cancer Discov. 2021;11(10):2506‐2523. 33972311 10.1158/2159-8290.CD-20-1285 PMC8487926

[95] Gerhard GM , Bill R , Messemaker M , Klein AM , Pittet MJ . Tumor‐infiltrating dendritic cell states are conserved across solid human cancers. J Exp Med. 2021;218(1):e20200264. 10.1084/jem.20200264 PMC7754678 33601412

[96] Liu Y , Zhang Q , Xing B , et al. Immune phenotypic linkage between colorectal cancer and liver metastasis. Cancer Cell. 2022;40(4):424‐437.e425. 35303421 10.1016/j.ccell.2022.02.013

[97] Pombo Antunes AR , Scheyltjens I , Lodi F , et al. Single‐cell profiling of myeloid cells in glioblastoma across species and disease stage reveals macrophage competition and specialization. Nat Neurosci. 2021;24(4):595‐610. 33782623 10.1038/s41593-020-00789-y

[98] Chen YP , Yin J‐H , Li W‐F , et al. Single‐cell transcriptomics reveals regulators underlying immune cell diversity and immune subtypes associated with prognosis in nasopharyngeal carcinoma. Cell Res. 2020;30(11):1024‐1042. 32686767 10.1038/s41422-020-0374-x PMC7784929

[99] Mulder K , Patel AA , Kong WT , et al. Cross‐tissue single‐cell landscape of human monocytes and macrophages in health and disease. Immunity. 2021;54(8):1883‐1900.e1885. 34331874 10.1016/j.immuni.2021.07.007

[100] Zernecke A , Erhard F , Weinberger T , et al. Integrated single‐cell analysis based classification of vascular mononuclear phagocytes in mouse and human atherosclerosis. Cardiovasc Res. 2022. 10.1093/cvr/cvac161 PMC10325698 36190844

